# Supplementary material for: Haplotype-aware long-read error correction
Source: Algorithms Mol Biol. 2026 Jun 27;21:13. doi: 10.1186/s13015-026-00302-3 (PMC13576334; doi:10.1186/s13015-026-00302-3)
Supplement: Supplementary file 1 [file 13015_2026_302_MOESM1_ESM.pdf]

Supplementary Document

Barak et al. 'Haplotype-aware Long Read Error Correction'

Contents

A Hardness result: full exposition 1

B Additional results 3

C Software versions and commands 7

Appendix A Hardness result: full exposition

Lemma 7 *The NP-hardness of Problem 3 implies NP-hardness of Problem 1*

Proof Our proof uses a chain of arguments. We begin by defining Problem 8, a simplified version of Problem 1 where we have a single term in the objective function and a binary alphabet. Next, we introduce Problem 9, a modified version of Problem 3. To prove the lemma, we first prove Claim 10 to show that Problem 9 is NP-hard, then Claim 11 to establish polynomial-time equivalence between Problem 9 and Problem 8. Finally we complete the proof by proving Claim 12.

Problem 8 *Given a set S of n vectors in $\{0,1\}^d$ and a positive integer $k \leq n$, compute a subset $S' \subseteq S$ of cardinality k and a center c in $\{0,1\}^d$ such that $\sum_{x \in S'} agree(c, x)$ is maximum.*

Problem 9 *Given a set S of n vectors in $\{-1,1\}^d$ and a positive integer $k \leq n$, compute a subset $S' \subseteq S$ of cardinality k that maximizes the ℓ_1 norm of the vector sum, i.e. $\left\|\sum_{x \in S'} x\right\|_1$.*

Claim 10 *The NP-hardness of Problem 3 (Lemma 5) implies the NP-hardness of Problem 9.*

Proof Problem 9 differs slightly from Problem 3 because we seek a subset of size exactly k in Problem 9, whereas we seek a subset of size at least k in Problem 3. We prove Claim 10 using a simple polynomial-time reduction from Problem 3 to Problem 9. Given an instance of Problem 3 with $S = \{x_1, \ldots, x_n\}$ and parameter $k = \alpha$, we construct $n - \alpha + 1$ instances for Problem 9 using the same set of vectors S and parameter $k = \alpha + i$, for all $0 \leq i \leq n - \alpha$. The maximum of the solution over all instances of Problem 9 is the solution to Problem 3. □

Claim 11 *Problems 8 and 9 are polynomial-time equivalent problems.*

Proof We consider a solution set of vectors $S' \subseteq \{0,1\}^d$ for Problem 8. Observe that the vector c must satisfy $c[i] = \text{majority}\left(\{x[i] \mid x \in S'\}\right)$, where ties are broken arbitrarily. Let $\phi : \{0,1\} \to \{-1,1\}$ be the coordinate-wise transformation defined by $\phi(0) = 1$ and $\phi(1) = -1$. For any vector $x \in \{0,1\}^d$, let $\hat{x} = \phi(x)$ denote its image

in $\{-1,1\}^d$. Let $\hat{S'} \subseteq \{-1,1\}^d$ be the set of vectors S' with ϕ applied to each vector. The objective function of Problem 8 can be written as

$$\sum_{x\in S'} agree(x,c) = \sum_{\hat{x}\in\hat{S'}} \frac{1}{2}\left(d + \sum_{i=1}^{d} \hat{x}[i]\cdot\hat{c}[i]\right) = \frac{1}{2}\left(kd + \sum_{i=1}^{d}\sum_{\hat{x}\in\hat{S'}} \hat{x}[i]\cdot\hat{c}[i]\right) = \frac{1}{2}\left(kd + \left\|\sum_{\hat{x}\in\hat{S'}} \hat{x}\right\|_1\right).$$

Thus, maximizing the objective function of Problem 8 is equivalent to maximising $\left\|\sum_{\hat{x}\in\hat{S'}} \hat{x}\right\|_1$.

Given a solution to Problem 8, i.e., a vector $c \in \{0,1\}^d$ and subset of vectors $S' \subseteq \{0,1\}^d$ that maximizes $\sum_{x\in S'} agree(x,c)$, we obtain a solution for Problem 9 by applying $\phi(\cdot)$ component-wise to every vector in S', creating the set $\hat{S'} \subseteq \{-1,1\}^d$. By our earlier argument, $\left\|\sum_{\hat{x}\in\hat{S'}} \hat{x}\right\|_1$ is maximised. Conversely, given a solution for Problem 9 of the form $\hat{S'} \subseteq \{-1,1\}^d$, we apply ϕ^{-1} to every vector in $\hat{S'}$ to obtain a solution $S' \subseteq \{0,1\}^d$ for Problem 8. The center vector c is obtained by taking a majority over each coordinate, and the objective function of Problem 8 (sum of agreement with c) is maximized. □

Claim 12 *The NP-hardness of Problem 8 implies the NP-hardness of Problem 1.*

Proof We prove this by polynomial-time reduction from Problem 8 to Problem 1 defined on alphabet $\Sigma = \{0,1\}$ (since this can be bijectively mapped to $\{A,T\}$). Given an instance of Problem 8 with set $S = \{x_1,\ldots,x_n\}$ and parameter $k = \alpha$, construct n instances of Problem 1 where the ith instance $(t^i, S^i, k = \alpha - 1)$ is constructed by considering x_i as the target vector t^i, set $S^i = S \setminus x_i$, and parameter $k = \alpha - 1$. The maximum value solution of all n instances of Problem 1 is the solution to Problem 8. □

□

Lemma 13 *Given a fractional solution to the Max-Cut problem, there exists a rounding procedure that transforms it into an integral solution which is at least as large as that of the fractional solution.*

Proof Let $z_i \in [0,1]$ be the given fractional assignment of vertex v_i, for $i \in [1,|V|]$. The extent to which an edge (v_i,v_j) is cut is given by $|z_i - z_j|$. Accordingly, the cut size of the given fractional partitioning is given by $\sum_{(i,j)\in E} |z_i - z_j|$. Next, consider the following procedure to convert fractional assignment of vertices into an integral assignment. Let t be a threshold chosen uniformly at random from the interval $[0,1]$. Assign vertex v_i to partition V_1 if $z_i > t$ and assign it to partition V_2 otherwise. For any edge $(v_i,v_j) \in E$, the probability that it is cut is given by $|z_i - z_j|$ because this occurs precisely when $z_i \le t < z_j$. Hence, the expected value of the cut size of our integral solution is $\sum_{(v_i,v_j)\in E} |z_i - z_j|$, which is equal to the cut size of the fractional cut. This expectation guarantees the existence of at least one threshold $t^* \in [0,1]$ for which the corresponding integral solution has value at least as large as the given fractional solution. Finally, observe that it suffices to check at only $|V|+2$ candidate values for t: namely, at each $z_i \in \{z_1, z_2, \ldots, z_{|V|}\}$, as well as the endpoints 0 and 1. This is because the objective remains constant between any two consecutive values in the sorted sequence $z_1 < z_2 < \ldots < z_{|V|}$. Therefore, an optimal value t^* can be found in polynomial time by checking the solution at only these $|V|+2$ points. □

Remark: Lemma 13 was stated directly without a proof in [15].

Appendix B Additional results

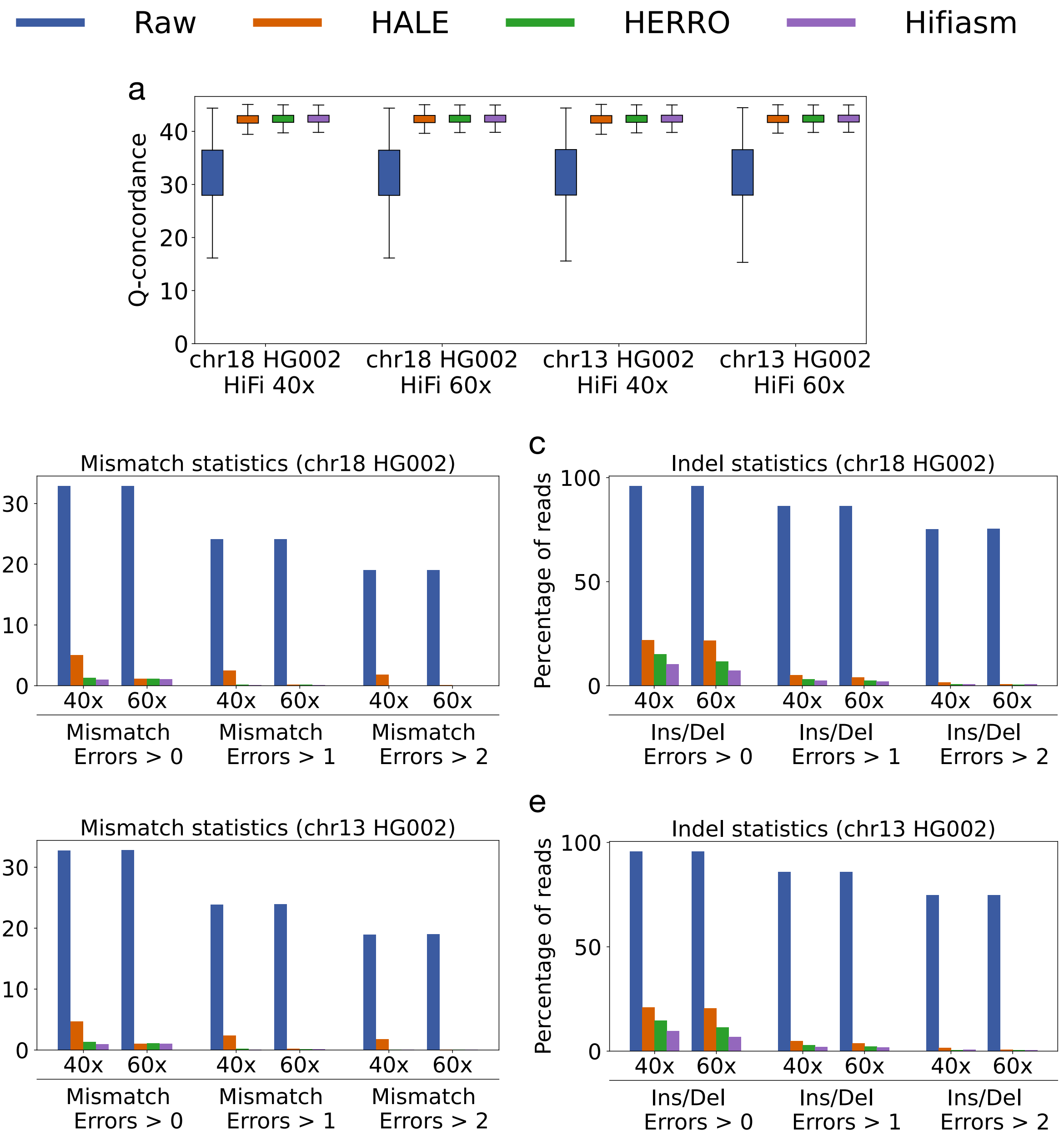

Fig. B1: Accuracy of sequencing reads from HG002 chromosomes 18 and 13, at 40× and 60× coverage. The plots compare the accuracy of raw reads with those corrected by HALE, Herro, and Hifiasm.

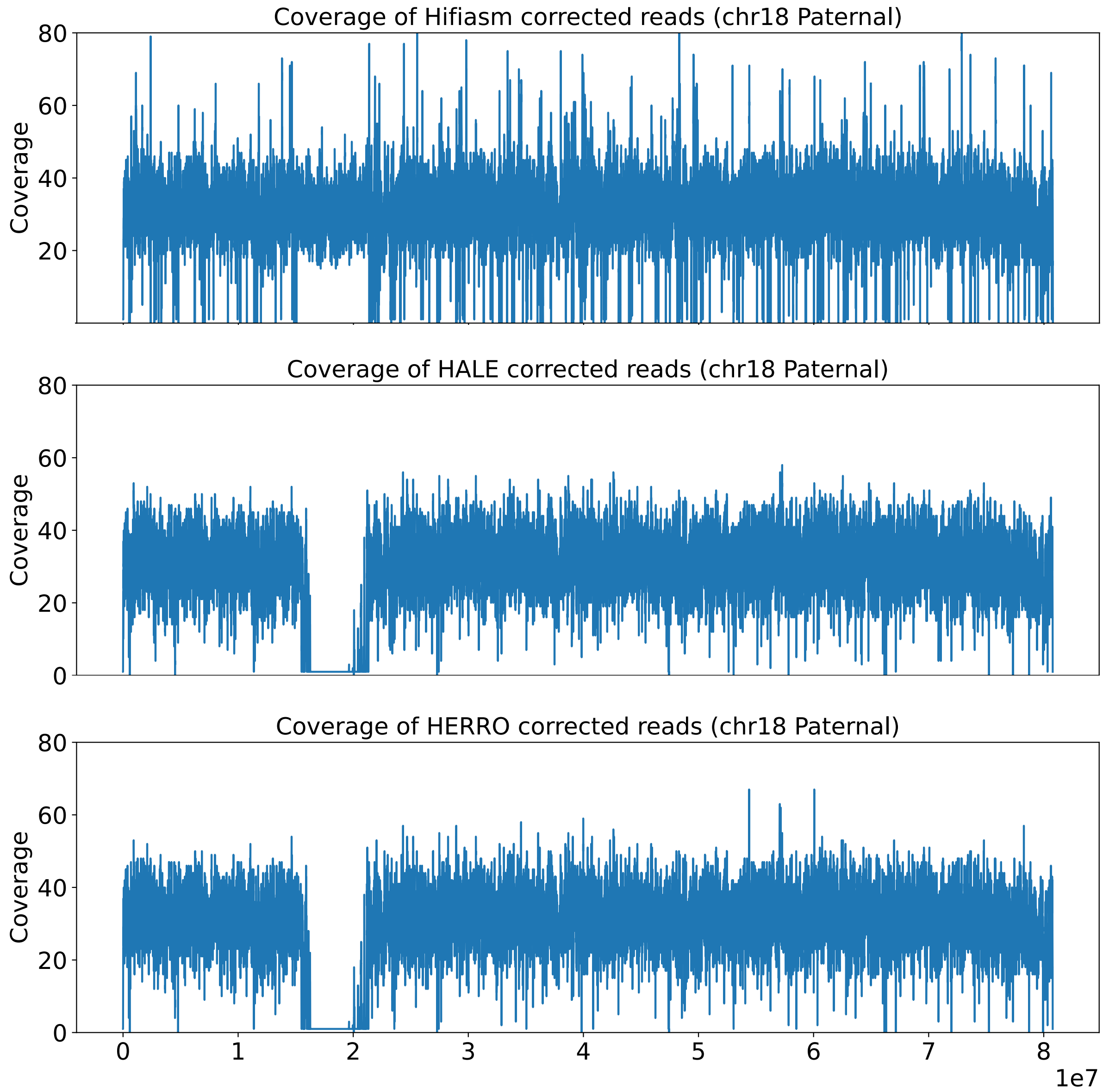

Fig. B2: Coverage of corrected reads using Hifiasm, HALE, and Herro, respectively, on human HG002 chromosome 18 (paternal). Note the drop in alignment coverage for HALE and Herro across the centromeric region of chromosome 18 (locus: 15,911,092 - 21,158,059). Both HALE and Herro rely on Minimap2 all-vs-all overlaps for read correction. Minimap2 produced insufficient alignments for reads originating from this region, causing both the methods to leave these reads uncorrected. We observed the same result in the maternal sequence as well (data not shown).

Dataset	Error type	# Errors	Percentage of reads			
			**Raw**	**HALE**	**Herro**	**Hifiasm**
HG002 chr9 40x	Mismatch	>0	31.3	3.7	1.6	1.3
		>1	22.7	1.9	0.7	0.6
		>2	17.6	1.4	0.6	0.6
	Indel	>0	95.6	22.6	14.9	9.7
		>1	85.8	4.9	3.1	2.5
		>2	74.9	1.6	1.1	1.3
HG002 chr9 60x	Mismatch	>0	33.4	1.9	1.2	1.0
		>1	24.3	0.5	0.2	0.1
		>2	19.1	0.3	0.0	0.0
	Indel	>0	96.0	23.1	13.2	7.9
		>1	86.9	5.1	2.7	2.0
		>2	76.3	1.3	0.6	0.7
A. thaliana 40x	Mismatch	>0	39.0	0.2	2.3	0.1
		>1	27.5	0.1	0.8	0.0
		>2	21.5	0.1	0.1	0.0
	Indel	>0	97.4	11.5	18.3	5.4
		>1	91.5	1.7	3.0	0.5
		>2	84.3	0.4	0.6	0.1
A. thaliana 60x	Mismatch	>0	39.0	0.2	1.9	0.2
		>1	27.5	0.1	0.6	0.1
		>2	21.6	0.1	0.1	0.0
	Indel	>0	97.4	12.3	22.7	3.9
		>1	91.5	1.8	4.5	0.4
		>2	84.3	0.4	0.9	0.1

Table B1: The exact values plotted in Figures 4b - 4e.

Dataset	Error type	# Errors	Percentage of reads			
			**Raw**	**HALE**	**Naive-1**	**Naive-2**
HG002 chr9 40x	Mismatch	>0	31.3	3.8	22.3	7.8
		>1	22.7	2.0	13.9	4.0
		>2	17.6	1.5	10.2	1.9
	Indel	>0	95.6	23.1	29.0	76.0
		>1	85.8	5.0	11.6	44.7
		>2	74.9	1.8	5.5	22.8
HG002 chr9 60x	Mismatch	>0	33.4	1.8	8.9	5.7
		>1	24.3	0.7	3.5	2.7
		>2	19.1	0.4	2.0	0.9
	Indel	>0	96.0	23.5	16.3	72.4
		>1	86.9	5.1	4.8	41.3
		>2	76.3	1.6	2.0	20.0
A. thaliana 40x	Mismatch	>0	39.0	0.2	0.3	3.7
		>1	27.5	0.1	0.1	1.6
		>2	21.5	0.1	0.1	0.4
	Indel	>0	97.4	11.4	6.7	68.8
		>1	91.4	1.8	1.0	40.2
		>2	84.3	0.5	0.4	21.9
A. thaliana 60x	Mismatch	>0	39.0	0.2	0.2	3.3
		>1	27.5	0.1	0.1	1.4
		>2	21.6	0.1	0.1	0.3
	Indel	>0	97.4	12.2	5.3	71.8
		>1	91.4	1.8	0.8	40.7
		>2	84.3	0.4	0.3	21.0

Table B2: The exact values plotted in Figures 5b - 5e.

Appendix C Software versions and commands

Tool	Version
SeqKit	2.5.1
Minimap2	2.26-r1175
Samtools	1.20
Hifiasm	0.25.0-r726
HALE	1.0.0
HERRO	Commit: 204d29e
Bamconcordance	Commit: dded267

Table C3: Versions of software used in our experiments.

<table>
<tr><th>Step</th><th>Command</th></tr>
<tr><td>Samtools (extract chr9 reads)</td><td>samtools view -b hg002_hifi_reads.bam chr9_MATERNAL chr9_PATERNAL | samtools fastq - > chr9_hg002_hifi_reads.fastq</td></tr>
<tr><td>SeqKit (read sampling)</td><td>seqkit sample -p <proportion> <input_fastq> -o <output_fastq></td></tr>
<tr><td>All-vs-all overlap</td><td>minimap2 -K8g -cx ava-ont -k25 -w17 -e200 -r150 -m2500 -z200 -f 0.005 -t 64 --dual=yes <reads> <reads></td></tr>
<tr><td>HERRO inference</td><td>./herro inference --read-alns <directory_alignment_batches> -t 8 -d 0,1,2,3 -m model_R10_v0.1.pt -b 64 <fastq.gz_input> <fasta_output></td></tr>
<tr><td>Hifiasm</td><td>./hifiasm -t 64 -o output_prefix.asm --write-paf --write-ec temp <fastq.gz_input></td></tr>
<tr><td>HALE</td><td>./hale inference --read-alns <directory_alignment_batches> -m "hale" -t 64 <fastq.gz_input> <fasta_output></td></tr>
<tr><td>Naive-1</td><td>./hale inference --read-alns <directory_alignment_batches> -m "consensus" -t 64 <fastq.gz_input> <fasta_output></td></tr>
<tr><td>Naive-2</td><td>./hale inference --read-alns <directory_alignment_batches> -m "pih" -t 64 <fastq.gz_input> <fasta_output></td></tr>
<tr><td>Mapping reads to reference</td><td>minimap2 -ax map-hifi --eqx -t 64 --secondary=no <reference> <fasta_input> | samtools view -@ 64 -S -b | samtools sort -@ 64 -o <bam_output> && samtools index -@ 256 <bam_output></td></tr>
<tr><td>BamConcordance (Using bam output from mapping step)</td><td>hg002-ccs/concordance/bamConcordance <fasta_input> <bam_output> <csv_output></td></tr>
</table>

Table C4: Commands used to run various tools.
